# Cytotoxicity and Antimicrobial Efficacy of Fe-, Co-, and Mn-Doped ZnO Nanoparticles

**DOI:** 10.3390/molecules29245966

**Published:** 2024-12-18

**Authors:** Hong Yin, Yang Lu, Rui Chen, Rebecca Orrell-Trigg, Sheeana Gangadoo, James Chapman, Ivan Cole, Vi Khanh Truong

**Affiliations:** 1 School of Engineering, RMIT University, Melbourne, VIC 3000, Australia; ivan.cole@rmit.edu.au; 2 Key Laboratory of Food Nutrition and Safety, Ministry of Education of China, College of Food Engineering & Biotechnology, Tianjin University of Science and Technology, Tianjin 300457, China; 3 Beijing Key Laboratory of Occupational Safety and Health, Institute of Urban Safety and Environmental Science, Beijing Academy of Science and Technology, Beijing 100054, China; chenrui@iuse.ac.cn; 4 School of Science, RMIT University, Melbourne, VIC 3000, Australia; s3486475@student.rmit.edu.au (R.O.-T.);; 5 School of Environment and Science, Griffith University, Nathan, QLD 4111, Australia; james.chapman@griffith.edu.au; 6 College of Medicine and Public Health, Flinders University, Bedford Park, Adelaide, SA 5042, Australia

**Keywords:** doped zinc oxide, nanoparticles, antimicrobial properties, *E. coli*, cytotoxicity

## Abstract

Zinc oxide nanoparticles (ZnO NPs) are one of the most widely used nanoparticulate materials due to their antimicrobial properties. However, the current use of ZnO NPs is hindered by their potential cytotoxicity concerns, which are likely attributed to the generation of reactive oxygen species (ROS) and the dissolution of particles to ionic zinc. To reduce the cytotoxicity of ZnO NPs, transitional metals are introduced into ZnO lattices to modulate the ROS production and NP dissolution. However, the influence of the doping element, doping concentration, and particle size on the cytotoxicity and antimicrobial properties remains unexplored. This study presents a comprehensive investigation of a library of doped ZnO NPs to elucidate the relationship between their physicochemical properties, antimicrobial activity against *Escherichia coli* (*E. coli*), and cytotoxicity to mammalian cells. The library comprises 30 variants, incorporating three different dopant metals—iron, manganese, and cobalt—at concentrations of 0.25%, 1%, and 2%, and calcined at three temperatures (350 °C, 500 °C, and 600 °C), resulting in varied particle sizes. These ZnO NPs were prepared by low temperature co-precipitation followed by high-temperature calcination. Our results reveal that the choice of dopant elements significantly influences both antimicrobial efficacy and cytotoxicity, while dopant concentration and particle size have comparatively minor effects. High-throughput UV–visible spectroscopic analysis identified Mn- and Co-doped ZnO NPs as highly effective against *E. coli* under standard conditions. Compared with undoped ZnO particles, Mn- and Co-doping significantly increased the oxidative stress, and the Zn ion release from NPs was increased by Mn doping and reduced by Fe doping. The combined effects of these factors increased the cytotoxicity of Mn-doped ZnO particles. As a result, Co-doped ZnO particles, especially those with 2 wt.% doping, exhibited the most favourable balance between enhanced antibacterial activity and minimized cytotoxicity, making them promising candidates for antimicrobial applications.

## 1. Introduction

The current rise of antimicrobial resistance to existing antibiotic drugs requires the urgent development of improved antimicrobial agents to replace ineffective traditional drugs [1]. These new agents exist in many forms, including revised forms of existing drugs, such as the penicillin family [2], drug combinations, such as amoxicillin–clavulanic acid [3], metals, such as silver [4], liquid metals [5], and other inorganic materials, such as metal oxide and chalcogenide nanomaterials [6,7].

Metal oxides have emerged as an as promising alternatives for combating bacterial pathogens, such as *Escherichia coli* (*E. coli*), which is often used as a model Gram-negative bacterial pathogen in the study of new antimicrobials due to its long history of scientific analysis [6]. The antimicrobial performance of synthetic metal oxides strongly depends on their chemical composition and morphology [8], and different synthesis methods have been developed to maximize the antimicrobial potential whilst minimizing adverse cytotoxicity to mammalian cells [9,10]. Various forms of metal oxides, such as thin films, atomically thin layers, nanoparticles (NPs), nanorods, and heterogenous structures have been studied [11,12,13,14,15]. However, nanomaterials possess some limitations that hinder their effective antimicrobial functions due to their agglomeration, instability, and intrinsic toxicity in aqueous environments [16].

ZnO NPs are known for their outstanding antibacterial properties. However, safety concerns about ZnO NPs have gained significant attention. Numerous studies have shown that ZnO can affect the DNA replication and mitochondrial functions of cells, producing cytotoxicity [17,18,19,20]. Many physicochemical characteristics of ZnO NPs are directly related to their biosafety, including size, shape, chemical composition, surface charge, solubility, biological binding sites, and metabolic and excretion pathways [21,22,23].

Doping ZnO particles with transitional metals can influence their cytotoxicity [24]. The method of doping, dopant metal, and general morphology have a significant impact on the efficacy of the particles, with different dopant metals being able to increase or decrease antimicrobial activity [25,26]. Doping can increase the activity of ZnO NPs through the addition of different elements which can release reactive oxygen species (ROS), modulate metal ions leached into cells or media, or disrupt membrane structures leading to cell death. Depending on the particle size, different dopant metals and concentrations may be more effective than others. The synergy between particle size, dopant element, and composition is important to characterize to achieve the best antibacterial results and minimum cytotoxicity.

Herein, a library of doped ZnO NPs was established with different doping elements (iron, manganese, and cobalt), doping concentrations, and particle sizes, and their impacts on the cytotoxicity and antibacterial properties were assessed. This study describes the chemical composition and morphology of the particles and tests their antimicrobial activity against the model organism *E. coli* and their cytotoxicity to two different cell lines to determine the suitability of the particles as potential antimicrobial agents and aims to find an effective antibacterial agent that also has low biological toxicity.

## 2. Results and Discussion

### 2.1. Characterization of ZnO NPs

Thirty types of ZnO particles were used for antimicrobial and cytotoxicity screening in this study. They were varied in doping element (Fe, Mn, and Co), doping concentration (0.25%, 1%, and 2%), and calcination temperature (350 °C, 500 °C, and 600 °C).

As shown in our previous publication [27], the shape and size distribution of doped and undoped particles were nearly identical. However, the particle size of undoped ZnO NPs was larger than that of doped ones. By measuring the diameters of 100 particles, undoped ZnO NPs calcinated at 350 °C had an average particle size of 25.8 nm, while those of Fe-doped, Mn-doped, and Co-doped ZnO NPs were 12.2 nm, 16.5 nm, and 17.0 nm, respectively (Figure S1). As reported by Sahu et al. [28], the shrinkage of particle size confirmed the successful doping because the doped atoms in the ZnO lattice inhibited the grain growth.

**Figure 1:**
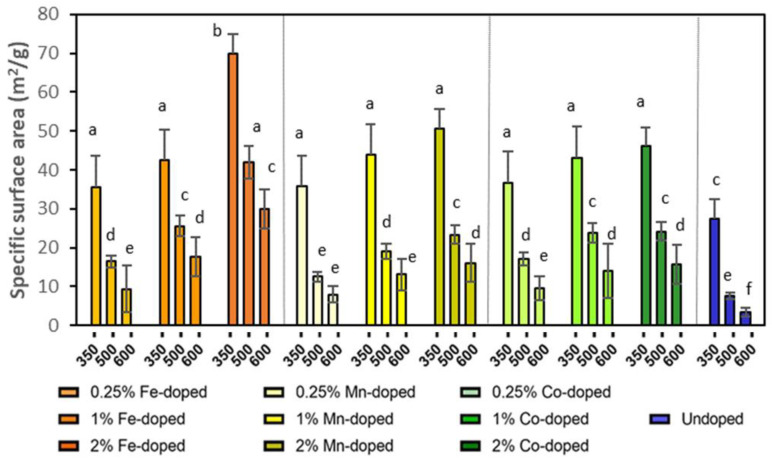
Specific surface area of doped and undoped ZnO NPs calcinated at 350 °C, 500 °C, and 600 °C. Different small letters indicate statistical difference from each other with *p* < 0.05.

The specific surface areas of doped and undoped ZnO NPs are compared in Figure 1. A higher calcination temperature was associated with a smaller specific surface area, i.e., a larger particle size. For undoped particles, the specific surface area decreased from 27.4 to 3.4 m^2^/g by increasing the calcination temperature from 350 to 600 °C. At each calcinated temperature, the specific surface areas of doped ZnO particles were larger than those of undoped particles. This was also attributed to the presence of doping atoms that inhibited grain growth during calcination, thus reducing the particle size and increasing the specific surface area. Moreover, when the doping concentration increased from 0.25% to 1% to 2%, the specific surface area increased for all doped NPs because more doping elements enhanced the inhibition effect and reduced the particle size further.

Doped particles were prepared by introducing a normal weight concentration of 0.25% to 1% to 2% doping element in the synthesis. Element analysis on the as-synthesised ZnO NPs suggested that the actual doping concentration was different from the nominal value (Table 1). For example, the Fe concentration in the NPs was slightly higher than the nominal weight concentration used in synthesis, while the doped Co was more than half of the nominal weight concentration. In contrast, Mn was difficult to incorporate into the ZnO lattice, with the doped concentration less than half of the nominal concentration. This finding can be related to the larger divergence in ionic sizes between Mn^2+^ (0.80 Å) and Zn^2+^ (0.74 Å). Comparatively, Fe^2+^ and Co^2+^ with ionic diameters comparable to that of Zn^2+^ (0.74 Å and 0.72 Å, respectively) allow an easy substitution.

X-ray diffraction (XRD) patterns (Figure S2) suggested that hexagonal wurtzite ZnO was the only phase identified in the doped and undoped NPs. No secondary phase was detected in the doped ZnO NPs. This could be attributed either to the low doping concentrations or to the incorporation of Mn, Co, or Fe as substitutional elements within the ZnO lattice. The local atomic environment of ZnO NPs was investigated using extended X-ray absorption fine structure (EXAFS), with the results reported in our previous publications [27]. It was concluded that that Co ions could substitute for Zn^2+^ sites in the lattice more completely because Co ions induced less short-range order changes in the ZnO structure than the incorporation of Mn or Fe. In addition, all of the particles had positive surface charge in water with zeta potentials in the range of 10–20 mV. The UV spectra of undoped and 2% Fe-, Co-, and Mn-doped ZnO NPs calcinated at 350 °C are shown in Figure S3. Based on the UV spectra, the band gap energies of the 2% doped ZnO were 3.33 eV, 3.32 eV, and 3.35 eV for Fe, Mn, and Co doping, respectively, which were not significantly different from that of undoped ZnO (3.33 eV).

### 2.2. Antibacterial Properties

**Figure 2:**
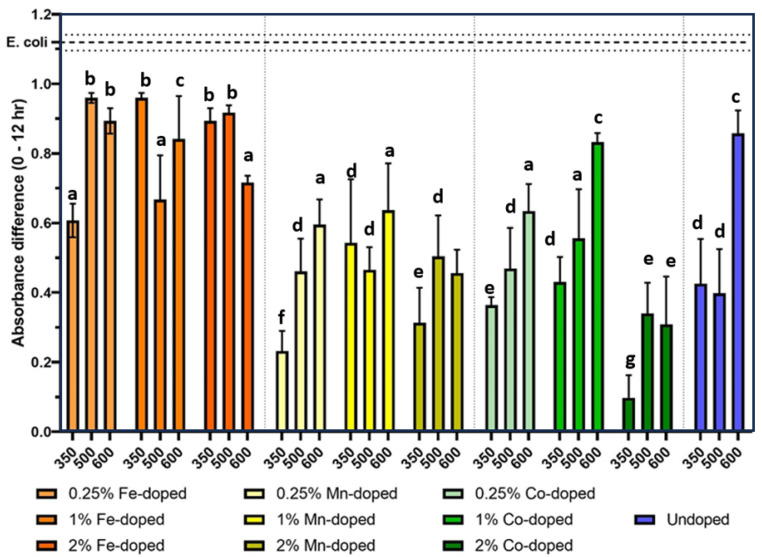
Absorbance difference between control *E. coli* samples (untreated) and *E. coli* samples treated with 50 mg/L of doped and undoped ZnO NPs for 12 h. Error bars denote standard error using four replicates per sample. Different small letters indicate statistical difference from each other with *p* < 0.05.

Figure 2 shows the antibacterial performance of thirty types of ZnO NPs at an exposure concentration of 50 µg/mL, which suggests several trends across dopant metal, doping concentration, and calcination temperature. First, undoped particles were effective in inhibiting *E. coli* growth at this concentration. The undoped NPs calcinated at 350 °C and 500 °C had similar antibacterial performance, while the particles calcinated at 600 °C were less effective possibly because their smaller specific surface area provided less active surface to contact with the bacteria. Compared to the undoped ZnO NPs, Fe-doped ones showed inferior antibacterial properties. Mn- and Co-doped ZnO NPs, however, were slightly more effective in inhibiting *E. coli* growth. Compared with the doping element, the doping concentration and calcination temperature (size and specific surface area) had insignificant impact. Among all 30 types of NPs, the 2% Co-doped ZnO NPs had the best antibacterial performance.

The growth curve of Fe-doped, Mn-doped, Co-doped, and undoped NPs are shown in Figures S4–S7, respectively, in comparison with those of *E. coli* and Luria–Bertani (LB) controls. The samples were measured every 30 min for 12 h, and the obtained absorbance values were normalized to the value at t = 0 h. For *E. coli*, the usual sigmoidal growth was observed in the absence of any ZnO NPs. Mn- and Co-doped ZnO NPs demonstrated lower normalized absorbance than Fe-doped and undoped ZnO, which was consistent with the inhibition at 12 h shown in Figure 2. In particular, 2% Co-doped ZnO NPs regardless of their calcination temperature (surface areas) had the best antibacterial performance, and their growth curves reached a maximum value (which was significantly lower than those produced by other ZnO particles) then declined further to the level of the LB control after 7 h of incubation.

### 2.3. Cytotoxicity

Based on the cell viability data of human umbilical vein endothelial cells (HUVECs), LC50 values (the concentrations of NPs that kill 50% of the tested cells) were extracted for cell toxicity evaluation. The LC50 values for NPs doped with different elements (Fe, Mn, and Co) are summarized in Table 2. The mean LC50 values were 45, 40, and 48 mg/L and the standard deviations were four, seven, and eight for Fe-, Mn- and Co-doped NPs, respectively. The statistical analysis suggested that the *p* values were all larger than 0.05, implying that the LC50 datasets were not statistically different.

**Figure 3:**
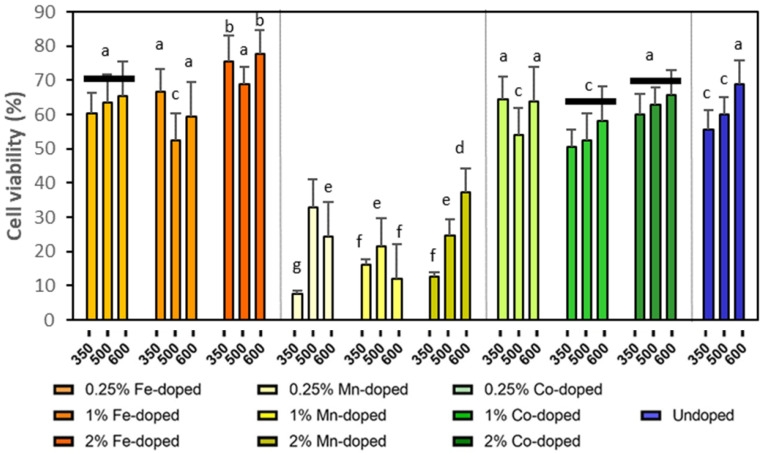
Cell viabilities of HepG2 cell line treated by 50 mg/L ZnO NPs doped with different elements and calcinated at different temperatures. Different small letters indicate statistical difference from each other with *p* < 0.05.

The above results showed that HUVECs were not sensitive to the characteristic changes in ZnO NPs. To further identify the cytotoxicity trend of doped ZnO particles, Figure 3 plots the cell viabilities of HepG2 cells treated by 50 mg/L of various ZnO NPs, which was the concentration used for the antibacterial testing.

For the undoped ZnO NPs, the cell viability increased with the calcination temperature, i.e., larger particle sizes corresponded to lower toxicity, which was consistent with our previous report [29]. After doping various elements, the size effect became less obvious. However, the cell viability solely depended on doping elements and followed the order of Co > Fe > Mn, which confirmed the above mentioned LC50 trend found in HUVECs (45, 40, and 48 mg/L for Fe-, Mn-, and Co-doped ZnO NPs, respectively). Our previous study demonstrated that Mn-doped ZnO were more toxic than Fe-doped and undoped NPs to WIL2NS cells [30], which was consistent with Figure 3. The cell viabilities of Co-doped NPs were similar to those of undoped NPs, suggesting that Co-doped NPs were less toxic than Mn-doped ones, but more toxic than Fe-doped ones.

The mechanism of NP toxicity is not specific to cells or to bacteria [31]. Two possible mechanisms for ZnO toxicity have been proposed, namely the production of ROS [32,33], and the release of Zn^2+^ ions [34,35]. In normal cell conditions, ROS are generated at low levels and are neutralized by antioxidant enzymes such as glutathione (GSH). Excessive levels of ROS can deplete GSH and accumulate oxidized GSH (GSSG), creating a condition of oxidative stress. Thus, cells react by mounting further protective or injurious responses. High ROS levels and oxidative stress have been identified as common reasons for cellular damage induced by NPs, including ZnO NPs. Excessive ROS can damage membranes directly or facilitate the internalization of NPs into cells and cause toxicity. In addition, ZnO NPs can release Zn^2+^ ions into the medium in a concentration-dependent manner. A strong correlation between ZnO NP-induced toxicity and free intracellular zinc concentration indicates a requirement for NP dissolution to precede cytotoxicity. The following sections analyse the contribution of each mechanism to the observed cytotoxicity and antibacterial properties of doped ZnO NPs.

### 2.4. Oxidative Stress

**Figure 4:**
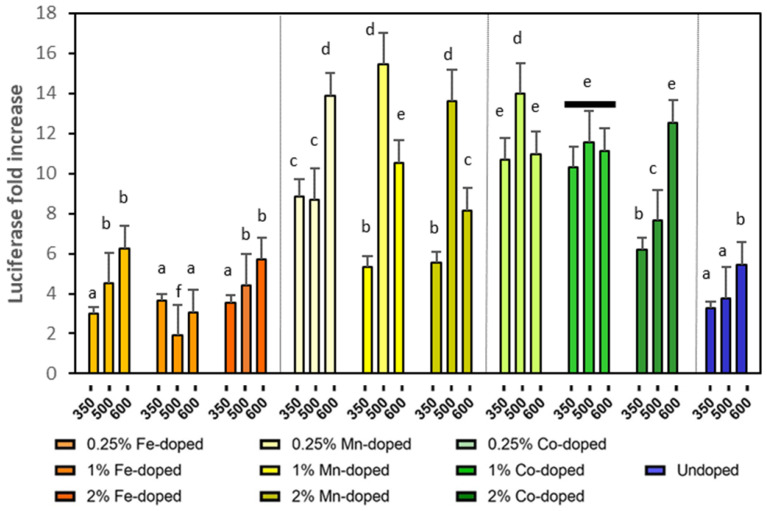
Highest luciferase fold increase in ZnO NPs with different doping elements calcinated at 350 °C, 500 °C, and 600 °C. Different small letters indicate statistical difference from each other with *p* < 0.05.

HepG2 antioxidant-response (ARE) reporter cell lines (HepG2-ARE) were used to assess the ability of oxidative stress production [23]. Luciferase assays were carried out and luciferase activities (response signal of oxidative stress) were expressed as fold induction relative to values obtained from untreated control cells. ARE expression increases as cells are stressed, but when they are severely damaged or dead, expression slows or ceases. The response to increasing concentrations of NPs (and oxidative stress) is therefore bell shaped rather than sigmoidal. The highest ARE values for each type of ZnO NP in the bell shape were summarized in Figure 4.

Generally, undoped ZnO NPs are not involved in electron reduction in biological systems because of the single oxidation state of Zn. However, there is evidence to suggest that cell death induced by ZnO NPs may be linked to the intracellular ROS generation [36]. In our study, the luciferase increases of undoped ZnO NPs remained at low levels, confirming that oxidative stress may not be the main reason for the toxicity of undoped ZnO [29].

Compared with undoped ZnO NPs, elevated oxidative stress was observed for Mn- and Co-doped NPs even when their actual doping concentrations were lower than that of Fe (Table 1). Consistent with our observation, several Mn- and Co-containing NPs also demonstrate abnormally high ROS levels. For example, doping 1.6 wt.% Mn in silica NPs could increase ROS by 25 folds in lung epithelial cells [37]. Furthermore, Co_3_O_4_ NPs led to significant ROS accumulation and DNA damage in a dose-dependent manner [38]. The different oxidation states of Mn or Co atoms could induce d–d electron exchanges and facilitate the surface redox activity. The presence of Mn and Co might also form defects, such as vacancies and holes, and affect the electronic distribution on the particles. In comparison to Co and Mn doping, Fe doping did not increase the ROS level of undoped ZnO NPs, despite the highest loading concentration of Fe (Table 1). The low ROS generation of Fe-doped ZnO could be associated with the suppressed NP dissolution, which will be discussed later. Therefore, the ROS production of doped ZnO was closely related to the doping elements, instead of the actual doping concentration.

### 2.5. Aqueous Solubility

The dissolution of doped ZnO NPs in deionized (DI) water was studied in comparison with that of undoped ZnO NPs. After 24 h of dissolution, only Zn was detected in the supernatant, and no doping elements (Fe, Mn, or Co) were detected by inductively coupled plasma-atomic emission spectroscopy (ICP-AES). The reason could be their very small doping concentration (0.25 to 2 wt.%).

**Figure 5:**
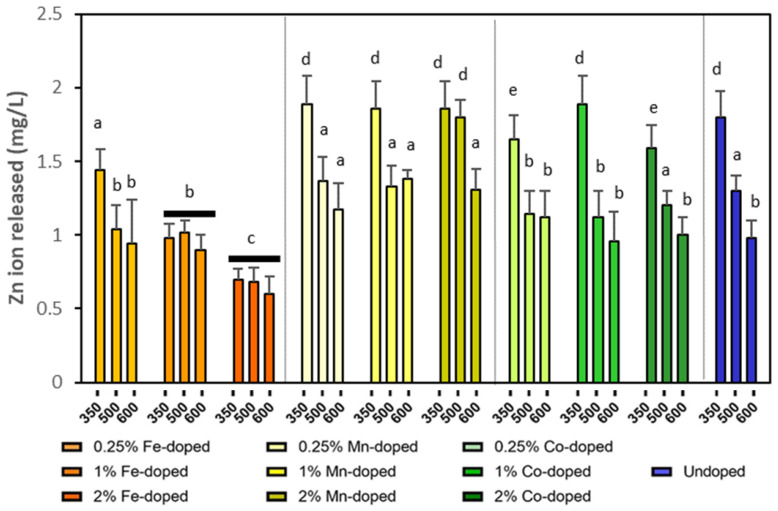
Released Zn ion concentrations from ZnO NPs with different doping elements calcinated at 350 °C, 500 °C, and 600 °C. Different small letters indicate statistical difference from each other with *p* < 0.05.

As shown in Figure 5, a smaller particle size (a lower calcination temperature) increased the released Zn ions because larger specific surface areas could contact DI water and facilitate the dissolution process. Therefore, the NPs calcinated at 350 °C generally had significantly higher ion release. For different doping elements, Fe-doped ZnO suppressed NP dissolution, and Co-doped ZnO had comparable solubility with undoped ZnO, while Mn-doped ZnO had slightly higher solubility than the undoped NPs, but the difference was only significant when the calcination temperature was 600 °C.

Consistent with our observation of the Fe-doped NPs having the lowest dissolution, a decreased dissolution rate and a low solubility of Fe-doped ZnO NPs had also been reported in 0.1 M sodium perchlorate solution at pH = 7 [39] and in seawater [40]. Fe substitution in a ZnO lattice might strengthen the binding to oxygen and limit proton-assisted dissolution, thus increasing the aqueous stability of Fe-doped NPs. The comparable dissolution behaviours of undoped and Mn-doped particles calcinated at lower temperatures were also consistent with the previous observation that Mn-doping calcinated at 350 °C did not affect the release of Zn ions in RPMI1640 medium [30].

Our research indicates that both the dissolved Zn ions and the ROS induced by transition metal doping are significant contributors to the toxic response of doped ZnO NPs. Therefore, when designing antibacterial ZnO NPs with low cytotoxicity, it is essential to consider them concurrently. Given the low solubility and ROS generation ability of Fe-doped NPs, these particles have low toxicity. Based on the same mechanism, their antibacterial properties were not as good as Mn- and Co-doped particles.

Co-doped ZnO NPs have excellent antibacterial performance (Figure 2). Their lower toxicity than Mn-doped particles is likely due to their more stable substitution structure (suggested by EXAFS study) and slightly less aqueous solubility (Figure 5). Therefore, Co-doped Zn NPs, especially those with 2% doping, were promising antibacterial agents due to their high bactericidal effect and low cytotoxicity.

## 3. Methods

### 3.1. Materials

All chemicals used in this study were purchased from Sigma-Aldrich (Castle Hill, NSW, Australia). DI water generated by a Milli-DI^®^ water purification system (Merck Millipore, Darmstadt, Germany) was used throughout the experiments.

### 3.2. Synthesis of ZnO NPs

For undoped ZnO NPs, Zn(CH_3_COO)_2_·2H_2_O (5.48 g) was dissolved in 50 mL of methanol to obtain a solution of 0.5 M. Then, a NaOH solution (1 mol/L, 1 mL) was added dropwise under 400 rpm magnetic stirring at 80 °C for 2 h. The precipitated white powders were centrifuged and re-dispersed in DI water until the pH of the supernatant reached 7. The powders were dried in a 120 °C oven overnight, followed by annealing at 350 °C (500 °C or 600 °C) for 2 h. Iron (II) acetate, manganese (II) acetate, and cobalt (II) acetate were added to the 50 mL of methanol as the transition metal precursors for the synthesis of doped ZnO NPs. The nominal concentrations of the doping elements were varied from 0.25 wt.% to 1 wt.% and 2 wt.%. Particle size was tuned by adjusting the calcination temperature from 350 °C to 500 °C and 600 °C. The actual doping concentration in the final powder was determined using ICP-AES (Varian Vista AX Simultaneous Axial, Melbourne, VIC, Australia) after the acid digestion of doped particles.

### 3.3. ZnO Characterization

A Bruker D8 Advance X-ray diffractometer (Bruker, Billerica, MA, USA) was employed to determine the crystalline phases of ZnO NPs. The particle morphologies were observed by a transmission electron microscope (TEM, JEOL, 100CX-II, Tokyo, Japan). Specific surface areas of the particles were determined by the Brunauer–Emmett–Teller (BET) gas-adsorption technique using a Micromeritics Tristar II 3020 surface area analyser (Micromeritics, Norcross, GA, USA). The surface charges of NPs suspended in DI water were measured with a Malvern Zeta Sizer 2000 (Malvern Panalytical, Westborough, MA, USA).

The solubility of ZnO NPs in DI water was measured as follows. ZnO NPs (5 mg) were dispersed into 50 mL of DI water in a plastic vial to obtain a ZnO concentration of 100 mg/L. The vial was then placed in a water bath shaker at room temperature. After shaking at 100 rpm for 24 h, 1.5 mL of the aliquots were taken from the suspension and centrifuged at 10,000 rpm for 30 min; 1 mL of the supernatant was added to 9 mL of DI water. The resulting zinc solution was analysed by ICP-AES to determine the released Zn concentration.

### 3.4. Antimicrobial Testing

*E. coli* ATCC 43888 were cultured in LB agar plates at 37 °C for 24 h to reach (1.4−5.0) × 10^6^ CFU/mL and added to LB broth prior to experiments. UV–visible spectroscopy was used to measure the effects of each doped ZnO NP on *E. coli* growth over 12 h. *E. coli* was adjusted to an optical density at 600 nm (OD_600_) of 0.1 in LB broth, and growth assays were performed in a 96-well plate using a CLARIOstar Plus Microplate Reader (BMG Lab Tech, Ortenberg, Germany). Three replicates for each ZnO NP were conducted by performing two 96-well plate experiments including 20 samples in each. Control samples of each ZnO NP in LB broth, LB media, and *E. coli* were also included in all plates. Assay plates were incubated at 37 °C in the microplate reader for 12 h. Bacterial growth was monitored over 12 h at 30 min intervals and spectra were taken automatically across 220–1000 nm at 2 nm resolution. The OD_600_ values were isolated from these spectra. UV–visible spectral data were exported from the CLARIOstar Plus Microplate Reader software (MARS) in *.csv format to Unscrambler (Version 11, Camo Analytics AS, Oslo, Norway) for chemometric analysis. The spectra were pre-processed using the Savitzky–Golay transformation (second derivatives, 10-point symmetrical smoothing, and 2nd polynomial order).

### 3.5. Cytotoxicity Testing

Cell viabilities were determined by the Cell Counting Kit-8 (CCK-8, Dojindo Molecular Technologies, Tokyo, Japan) assay. Primary HUVECs at a confluence level of 10^4^ cells per well in a 96-well plate were exposed to doped and undoped ZnO NPs at concentrations from 0 to 200 µg mL^−1^ according to previously described methods [23]. HepG2 cells for the cytotoxicity test were seeded at 5 × 10^3^ cells per well in a 96-well plate and cultured at 37 °C in a 5% CO_2_ atmosphere in Dulbecco’s modified Eagle’s medium (DMEM, Gibco, Grand Island, NY, USA) supplemented with 10% foetal bovine serum (FBS, Gibco, USA). A HepG2-ARE cell line was constructed from a Cignal Lenti ARE Reporter assay kit (SABiosciences, Frederick, MD, USA) and used to evaluate the oxidative stress after exposure to the ZnO NPs [23].

### 3.6. Statistical Analysis

To compare different experimental groups, a one-way analysis of variance was performed using Prism 4.0 (GraphPad, Inc., San Diego, CA, USA) software. A value of *p* < 0.05 was considered to be statistically significant.

## 4. Conclusions

This study established a library of thirty undoped and doped ZnO NPs by adopting three dopant metals (iron, manganese, and cobalt), three dopant weight concentrations (0.25%, 1%, and 2%), and three calcination temperatures (350 °C, 500 °C, and 600 °C). Both cytotoxicity and antibacterial properties strongly depended on the doping element, while the doping concentration and calcination temperature, which led to various particle sizes and specific surface areas, had negligible effects. Mn- and Co-doped ZnO NPs demonstrated similar antimicrobial activity against *E. coli* as undoped particles. At the concentration used in the antimicrobial test (50 μg/mL), Co-doped NPs showed the lowest cytotoxicity while Mn-doped ones showed the highest cytotoxicity. As antimicrobial activity and mammalian toxicity must be considered concurrently when selecting particles for likely usage, Co-doped ZnO NPs are more promising antibacterial agents because of their excellent antibacterial performance and low cytotoxicity.

## Figures and Tables

**Table 1:** Nominal and actual doping concentration of doping elements in ZnO NPs.

Nominal Concentration (wt.%)	Fe (wt.%)	Mn (wt.%)	Co (wt.%)
0.25%	0.29	0.12	0.20
1%	1.14	0.36	0.64
2%	2.12	0.77	1.12

**Table 2:** LC50 values (mg/L) for ZnO NPs doped with different materials calcinated at different temperatures after exposure to HUVECs.

Temperature (°C)	Undoped	0.25% Fe	1% Fe	2% Fe	0.25% Mn	1% Mn	2% Mn	0.25% Co	1% Co	2% Co
350	50	40	43	50	32	51	38	43	45	43
500	55	45	41	45	40	36	50	44	68	45
600	60	48	40	50	40	31	42	54	45	48

## Data Availability

The original contributions presented in this study are included in the article/Supplementary Material. Further inquiries can be directed to the corresponding authors.

## Supplementary Materials

The following supporting information can be downloaded at: https://www.mdpi.com/article/10.3390/molecules29245966/s1.
