# Supplementary material for: Cytotoxicity and Antimicrobial Efficacy of Fe-, Co-, and Mn-Doped ZnO Nanoparticles
Source: Molecules. 2024 Dec 18;29(24):5966. doi: 10.3390/molecules29245966 (PMC11678603; doi:10.3390/molecules29245966)
Supplement: Supplementary file 1 [file molecules-29-05966-s001.zip › molecules-3348815-supplementary.pdf]

Supporting Information for

Cytotoxicity and Antimicrobial Efficacy of Fe-, Co-, and Mn-Doped ZnO Nanoparticles

Hong Yin [1,*], Yang Lu [2], Rui Chen [3], Rebecca Orrell-Trigg [4], Sheeana Gangadoo [4], James Chapman [5], Ivan Cole [1] and Vi Khanh Truong [6,*]

[1] School of Engineering, RMIT University, Melbourne, VIC 3000, Australia; ivan.cole@rmit.edu.au
[2] Key Laboratory of Food Nutrition and Safety, Ministry of Education of China, College of Food Engineering & Biotechnology, Tianjin University of Science and Technology, Tianjin 300457, China; luyang@tust.edu.cn
[3] Beijing Key Laboratory of Occupational Safety and Health, Institute of Urban Safety and Environmental Science, Beijing Academy of Science and Technology, Beijing 100054, China; chenrui@iuse.ac.cn
[4] School of Science, RMIT University, Melbourne, VIC 3000, Australia; s3486475@student.rmit.edu.au (R.O.-T.); sheeana.gangadoo@rmit.edu.au (S.G.)
[5] School of Environment and Science, Griffith University, Nathan, QLD 4111, Australia; james.chapman@griffith.edu.au
[6] College of Medicine and Public Health, Flinders University, Bedford Park, Adelaide, SA 5042, Australia
***** Correspondence: hong.yin@rmit.edu.au (H.Y.); vikhanh.truong@flinders.edu.au (V.K.T.)

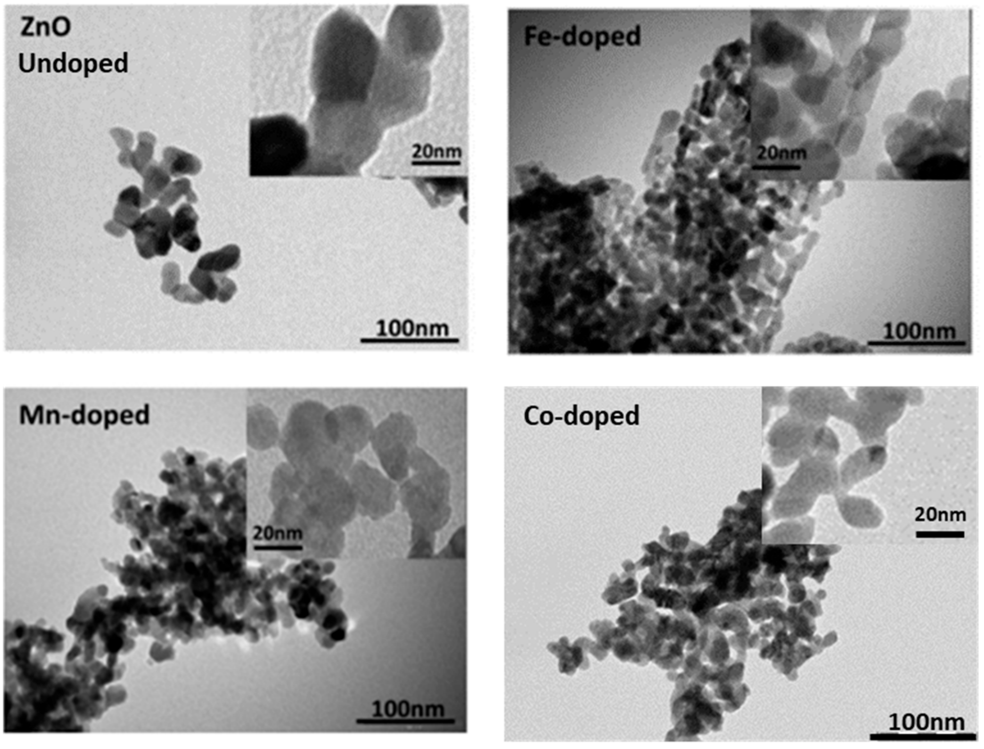

Figure S1 TEM images of undoped ZnO NPs and 2% Fe-, Co-, and Mn-doped ZnO NPs calcinated at 350°C. Insets show the magnified images of each sample.

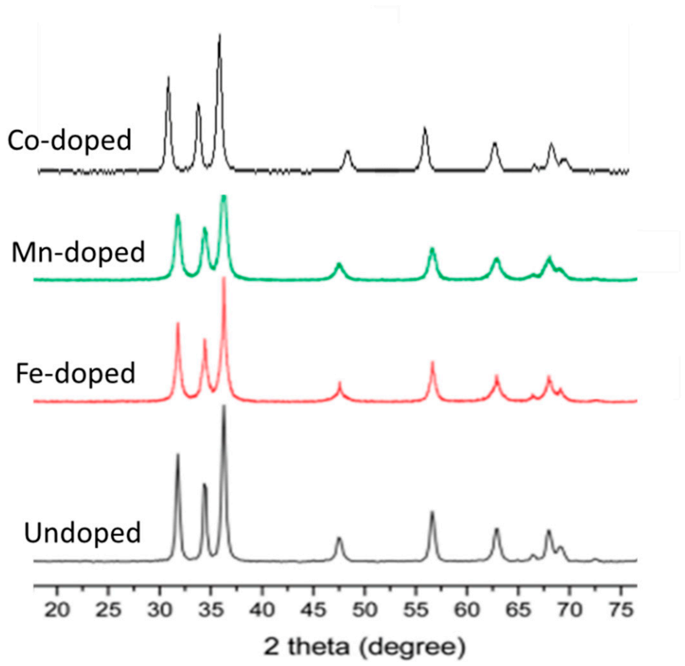

Figure S2 XRD patterns of undoped and 2% Fe-, Co-, and Mn-doped ZnO NPs calcinated at 350°C.

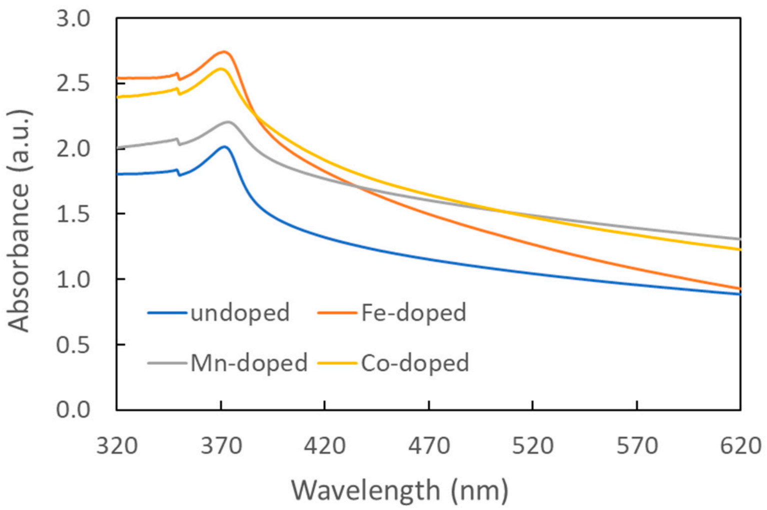

Figure S3 UV spectra of undoped and 2% Fe-, Co-, and Mn-doped ZnO NPs calcinated at 350°C.

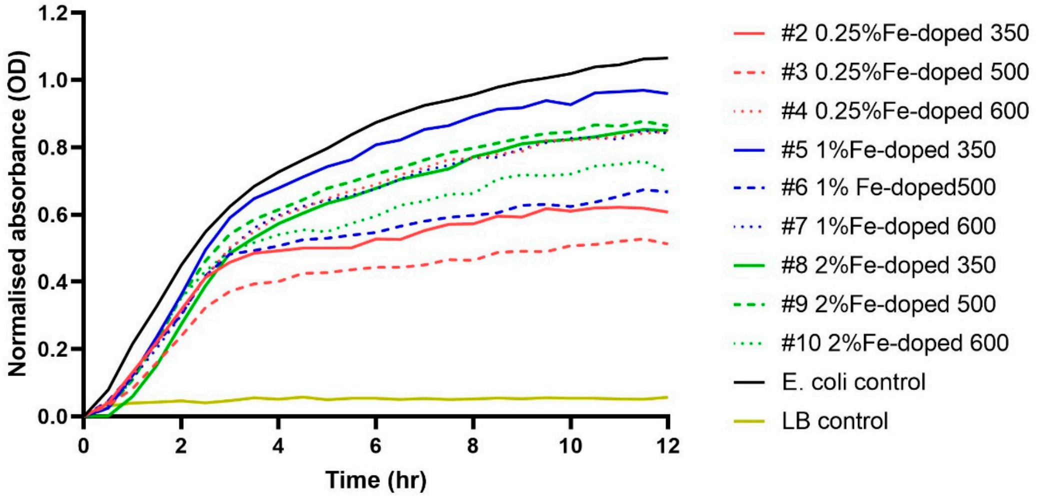

Figure S4 Normalized OD_{600} growth curves of Fe-doped ZnO NPs over 12 h. Absorbance values sampled every 30 min. Values normalized to set origin at OD_{600} value for each sample at t = 0 h.

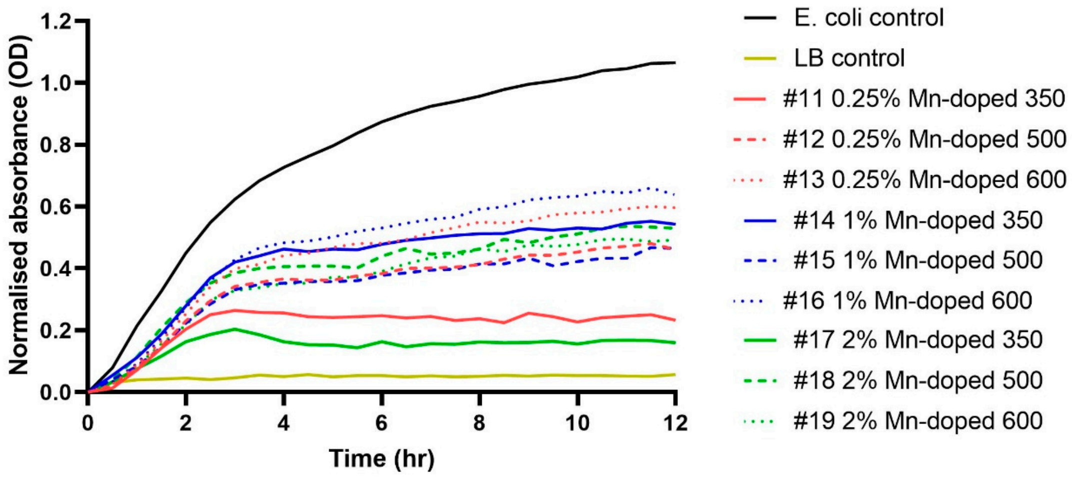

Figure S5 Normalized OD_{600} growth curves of Mn-doped ZnO NPs over 12 h. Absorbance values sampled every 30 min. Values normalized to set origin at OD_{600} value for each sample at t = 0 hr.

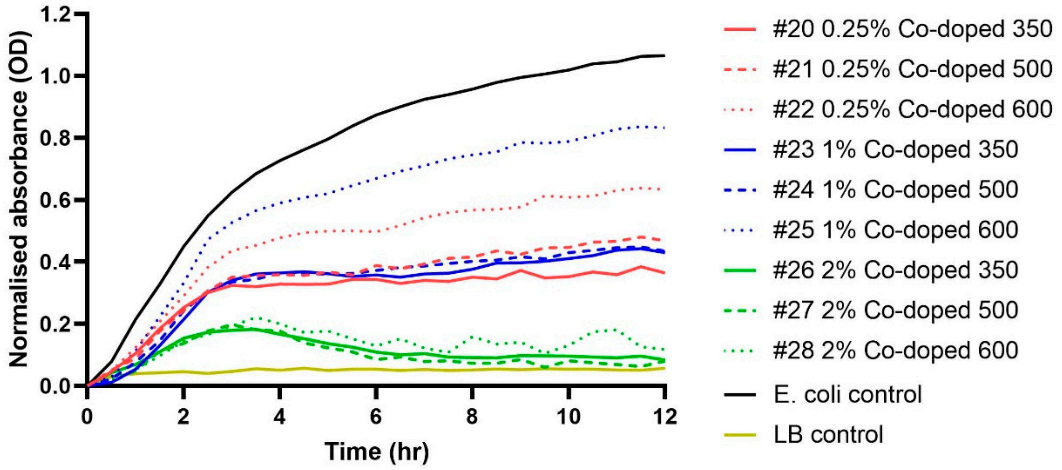

Figure S6 Normalized OD_{600} growth curves of Co-doped ZnO NPs over 12 h. Absorbance values sampled every 30 min. Values normalized to set origin at OD_{600} value for each sample at t = 0 h.

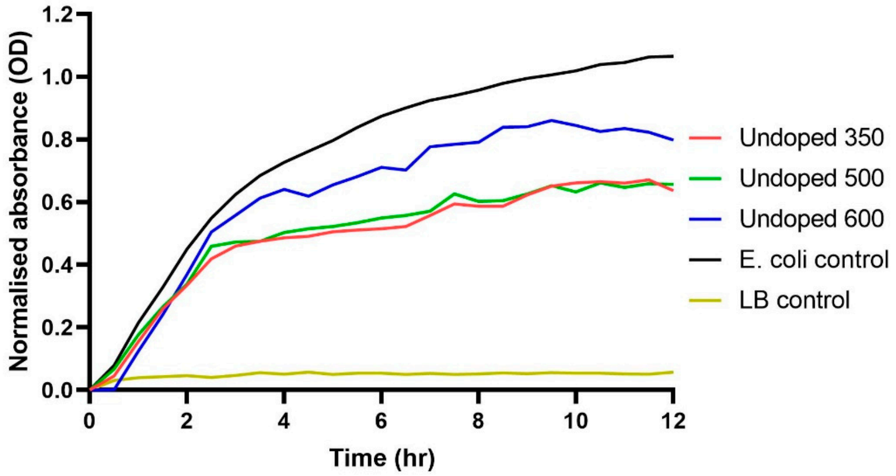

Figure S7 Normalized OD_{600} growth curves of undoped ZnO NPs over 12 h. Absorbance values sampled every 30 min. Values normalized to set origin at OD_{600} value for each sample at t = 0 h.
